# Development of Small Molecular Proteasome Inhibitors Using a *Caenorhabditis elegans* Screen

**DOI:** 10.1155/2014/237286

**Published:** 2014-11-11

**Authors:** Sudhir Nayak, Michela Fiaschi, Dana King, Erica R. Tabakin, Lyndsay Wood, David A. Hunt

**Affiliations:** ^1^Department of Biology, The College of New Jersey, 2000 Pennington Road, Ewing, NJ 08628, USA; ^2^Department of Chemistry, The College of New Jersey, 2000 Pennington Road, Ewing, NJ 08628, USA

## Abstract

We have developed a screening protocol to identify compounds with characteristics of small molecule proteasome inhibitors using the real-time analysis of the *Caenorhabditis elegans* germ line. This screen is able to identify compounds that induce germ line phenotypes characteristic of a reduction in proteasome function such as changes in polarity, aberrant nuclear morphology, and stimulation of apoptosis. This basic protocol is amenable to a high throughput (96-well) format and has been used successfully to identify multiple compounds for further analysis based on structural elements from the naturally occurring compounds lactacystin and the *β*-lactone homologs omuralide and salinosporamide A. The further development of this assay system should allow for the generation of novel small molecule proteasome inhibitors in a genetically tractable whole animal amenable to biochemical analysis.

## 1. Introduction

The controlled turnover of proteins is essential for the majority of cellular processes, including cell proliferation and cell death. The bulk of protein turnover in the cell is governed by the 26S proteasome, a highly conserved multisubunit protease complex with essential roles in regulating proteins levels in the cytoplasm and nucleus of all eukaryotes [1–5]. In addition to the well-studied roles of 26S in cell cycle regulation and removal of misfolded proteins, proteasome activity has also been implicated stress response, gene expression, DNA repair, immune regulation, and carcinogenesis [6].

**Figure 1:**
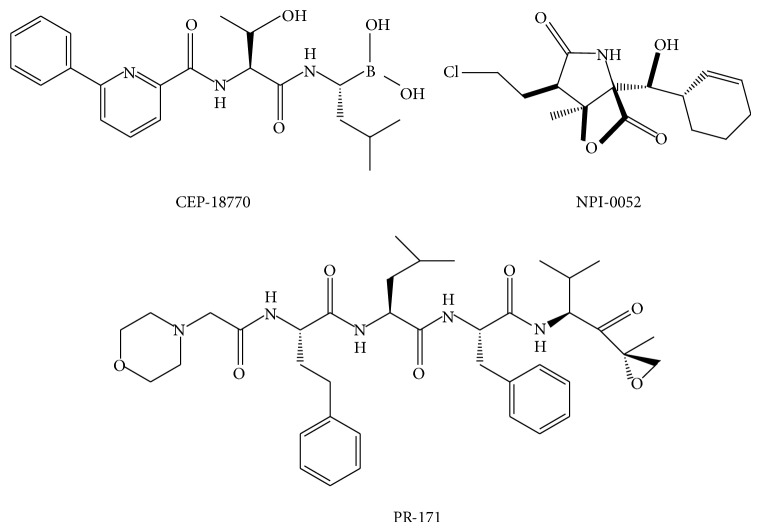


The central role of the 26S proteasome in the selective degradation of intracellular proteins involved in the cell cycle has made it a target of considerable interest in the development of novel anticancer therapeutics. Over the last 15 years, a variety of synthetic and natural compounds have been characterized that selectively inhibit the proteasome and fall into seven major categories (aldehydes, *β*-lactones, epoxyketones, boronates, vinyl sulfones, cyclic peptides, and macrocyclic vinyl ketones) [7]. Other than cyclic peptides, both synthetic and natural inhibitors have a small molecule backbone with a reactive pharmacophore that is able to interact with the N-terminal threonine of the catalytic *β*-subunits and interfere with proteolytic activity [8]. All categories of synthetic and natural proteasome inhibitors have been useful in elucidating the role of the 26S proteasome in normal cellular processes. Importantly, the dipeptidyl boronic acid Bortezomib (MG-341, PS-341, Velcade) has also been approved as a therapeutic in the treatment of multiple myeloma [9]. The success of Bortezomib has resulted in a considerable amount of interest in the development of small molecule anticancer therapeutics that targets the proteasome with novel modes of action. For example, the newly developed inhibitors carfilzomib (PR-171), salinosporamide A (NPI-0052), and CEP-18770 are currently in clinical trials (Figure 1) [7].

It is clear that proteasome inhibition has been a successful treatment strategy for a variety of cancers. While effective, some limitations have been found with the currently available therapeutics that include resistance (both intrinsic and acquired), toxicity, and inadequate delivery to solid tumors, among others. It is critical to develop novel compounds that are able to circumvent these problems in order to improve proteasome inhibitor-based therapies. As such, the development of easily synthesized small molecules that inhibit this system will be important for both resolving the biological roles of the proteasome and as possible chemotherapeutic agents with further development in medicinal chemistry programs.

**Figure 2:**
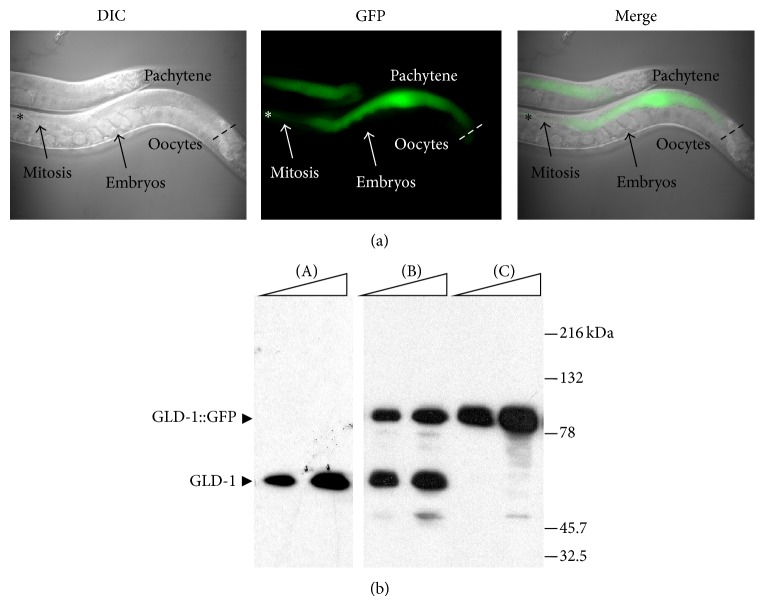
The* rrf-1(pk1417)(I);ozIs2(II)* (GLD-1::GFP) strain can be used to score GLD-1 levels in real-time. (a) DIC (differential interference contrast), GFP, and merged images of a live animal expressing GLD-1::GFP. The distal tip of the germ line is indicated by the “∗” and the dashed line is at the loop region (pachytene-diplotene/diakinesis boundary). (b) The expression levels of GLD-1::GFP are similar to endogenous GLD-1. Western blotting was performed on lysates of 25 and 50 worms of N2 ((A), wild-type),* rrf-1(pk1417)(I);ozIs2(II)* (B), and* gld-1(q485)(I);ozIs2(II)* (C). GLD-1 and GLD-1::GFP are indicated by the arrows. N2 has a single band at approximately 60 kDa (A). As expected, a strain carrying the* ozIs2* transgene in the* gld-1* null background only has the larger GLD-1::GFP fusion band at approximately 85 kDa (B). The* rrf-1(pk1417)(I);ozIs2(II)* has both bands from endogenous GLD-1 and the GLD-1::GFP fusion (C).

**Figure 3:**
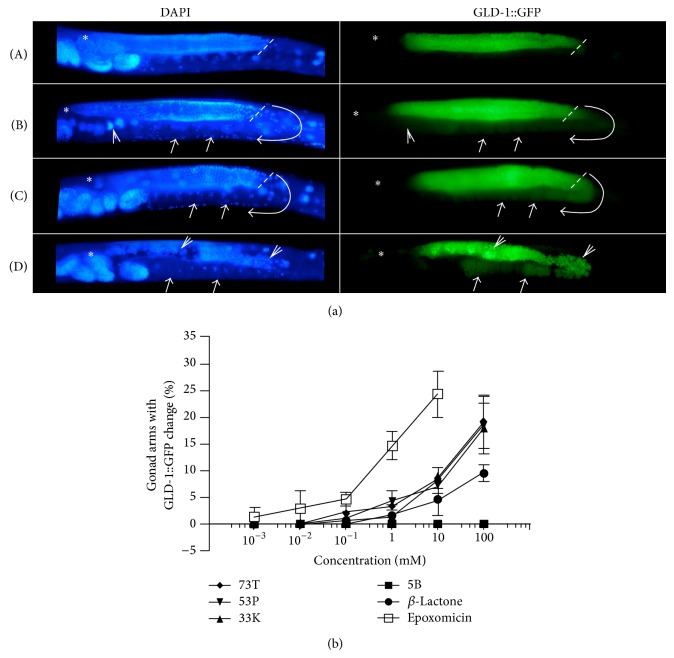
Screen using* rrf-1(pk1417)(I);ozIs2(II)*. (a) Paired DAPI (nuclei, left) and GLD-1::GFP (right) images from animals treated with carrier control (A) or compound 73T (B, C, D). Representative images of animals scored positive for GLD-1 misexpression are shown in panels B–D and quantified on the graph (bottom). The primary phenotypes associated with GLD-1::GFP were a transient increase in GLD-1::GFP (B), extension of into oocytes (C), and abnormal nuclear morphology coupled with ectopic GLD-1::GFP expression (D). For all images the “∗” = distal tip of the germ line (mitosis), dashed line is at the loop region (pachytene-diplotene/diakinesis boundary), curved line ending in an arrow follows the germ line, closed arrow heads are examples of ectopic GLD-1::GFP expression, and “Λ” indicates abnormal nuclei. All GFP exposure times were identical for control and test conditions and all images are from 6 hours of treatment. (b) Compounds 73T, 53P, 33K, 5B, clasto-lactacystin *β*-lactone, and epoxomicin were tested for their ability to cause the expression of GLD-1::GFP. Gonad arms with changes in GLD-1 expression were graphed as a faction of total arms scored (%) for each concentration. To illustrate the dispersion of the data, the bars indicated ± SD (standard deviation) of *n* = 4 samples for each point.

*Caenorhabditis elegans (C. elegans) *is underutilized as a system to screen for novel bioactive compounds.* C. elegans* has an invariant cell lineage; however, unlike the 959 terminally differentiated somatic cells, the hermaphrodite germ line contains actively dividing and differentiating cells [10–12].* C. elegans* hermaphrodites have two U-shaped gonad arms with mitotic cells confined to the region near the distal tip cell and successively later stages of meiosis more proximal to the uterus. Cells within germ line can be distinguished both by their position in the gonad arm and by their nuclear morphology using DAPI (4′,6-diamidino-2-phenylindole) staining of fixed specimens (Figures 2(a) and 3(a)) [13].

We took advantage of the central role that proteasome function plays in the* C. elegans* germ line to develop an assay for novel small molecule inhibitors amenable to high throughput screening. Similar to other metazoans,* C. elegans* contains orthologs of all 14*α* type and *β* type subunits that make up the 20S proteasome core and homologs of at least 18 components of the 19S proteasome regulatory complex [14]. Reflecting the essential function of the proteasome in* C. elegans*, RNAi depletion of core proteasome subunits causes F1 larval lethality and sterility in *P*_*o*_ adults [15, 16]. In addition to the essential roles described in other species, more subtle roles for the proteasome have been described in the regulation of entry into meiosis and germ line sex determination via genetic analysis in* C. elegans* [17, 18].

To facilitate rapid screening for compounds, we used a* C. elegans* strain carrying an integrated transgene with the germ line tumor suppressor GLD-1 (germ line defective) fused to GFP (green fluorescent protein). GLD-1 is a KH-domain RNA binding protein with more than 100 mRNA targets. The expression pattern of GLD-1 is tightly restricted and is involved in multiple aspects of germ line development including meiotic entry, progression through meiotic prophase, and oocyte differentiation [19–21]. Disruptions in the expression pattern of GLD-1 can lead to pachytene (meiotic prophase) progression defects that can result in germ line tumors, meiotic entry defects, and aberrant germ line sex determination [19, 20]. In addition, RNAi of SCF (Skp1-Cullin-F-box protein complex) components involved in the ubiquitin mediated degradation of proteins result in the ectopic accumulation of GLD-1 prior to disruptions in germ line morphology suggesting that it may be a substrate of the proteasome [22]. The critical role of GLD-1 in multiple aspects of germ line development allows the GLD-1::GFP transgene to function as a real real-time readout of both germ line polarity and overall germ line health.

In this report we describe a screening procedure for the identification of compounds with characteristics of proteasome inhibitors using the real-time analysis of* C. elegans* germ line carrying a GLD-1::GFP transgene. We focused on the ability of compounds to phenocopy RNAi reduction-of-function germ line phenotypes of proteasome subunits and known proteasome inhibitors, such as altering the expression of a germ line transgene, disruption of nuclear morphology, and induction of apoptosis. The basic protocol has been scaled to 96-well format and has been used to identify multiple novel small molecules with characteristics of proteasome inhibitors based on structural elements from the naturally occurring compounds lactacystin [23] and the *β*-lactone homologs omuralide [24] and salinosporamide A [25]. Screening in 96-well format using whole animal model systems has some specific advantages. For example, we are able to screen a variety of cell types and rapidly eliminate compounds that cause resulted in lethality (toxicity), were not able to circumvent MDR transporters, or caused gross morphological changes unrelated to diving cells. Further development of this assay system should allow for the generation of novel small molecule proteasome inhibitors in a genetically tractable whole animal system that is also amenable to biochemical analysis.

## 2. Results and Discussion

### 2.1. Chemistry

**Figure 4:**
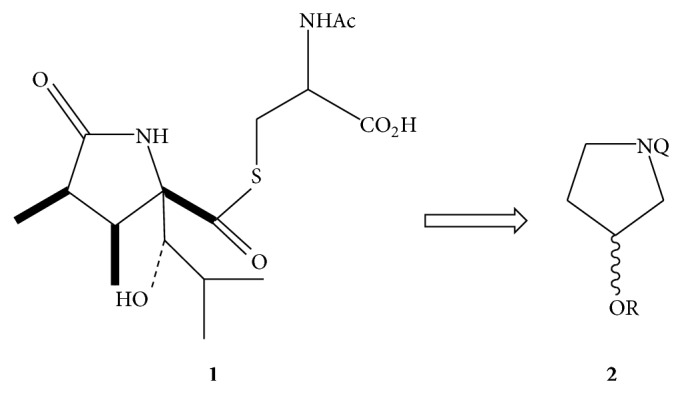


A set of small molecules of the general type** 2** bearing core structural similarities based on the natural product lactacystin (**1**) [23] and the *β*-lactone homologs omuralide (**3**) [24] and salinosporamide A (**4**) [25] were prepared (Figure 4) and screened in comparison to the known proteasome inhibitors epoxomicin (**5**) and omuralide. These potential inhibitors are readily derived from commercially available N-protected (benzyl and t-BOC) 3-hydroxypyrrolidine.

Studies pertaining to the structural requirements of the *γ*-lactam for biological activity have been described. Schreiber's report of the activity of lactacystin toward the 20S proteasome included a limited SAR study of lactacystin (**1**) and the related *β*-lactone omuralide** 3**, along with other analogs [24].

**Figure 5:**
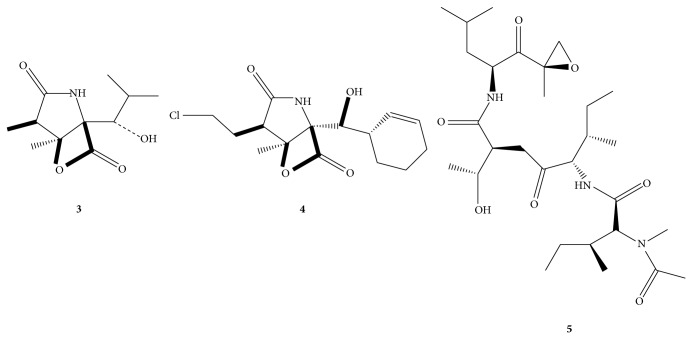


Inhibition studies revealed that the *γ*-lactam core and indicated stereochemistry of** 1** were required to observe activity. However, the marked increase in kinetic inhibition by the *β*-lactone (**3**, Figure 5) indicates the crucial nature of the electrophilic carbonyl group with regard to proteasome inhibition. The *β*-lactone exhibits activity that is 15-fold more than that of lactacystin, presumably due to the increased electrophilicity of the lactone carbonyl compared to the carbonyl of the thioester. Variations of the carboxylate function of the* N*-acetylcysteine side chain of lactacystin as well as deletion of the amide function had little effect on the activity of the analogs thereby confirming the presumption that the presence and reactivity of the C-4 carbonyl are crucial [25].

Corey's group studied the optimization of the C-7 alkyl and hydroxyisobutyl substituents of the *β*-lactone [26]. The hydroxyisobutyl side chain proved to be necessary for sufficient inhibition, resulting in compounds possessing ca. 10-fold greater biological activity compared to the most active analog known at that time. X-ray studies have revealed that the branched C-3 group of the hydroxyisobutyl side chain binds to a hydrophobic pocket of the lactacystin-labeled proteasome subunit [27]. Based on this information, C-7 analogs of the *β*-lactone demonstrated a remarkable increase of inhibition when with alkyl chain extensions. Inhibition rates more than doubled with the introduction of longer alkyl chains. Stereochemistry at C-7 likewise proved crucial, with the 7-epi analog resulting in a decrease in activity indicating that the original stereochemistry of the molecule is a requirement. Furthermore, kinetic inhibition data indicated the requirement of the *γ*-lactam core.

**Scheme 1:**
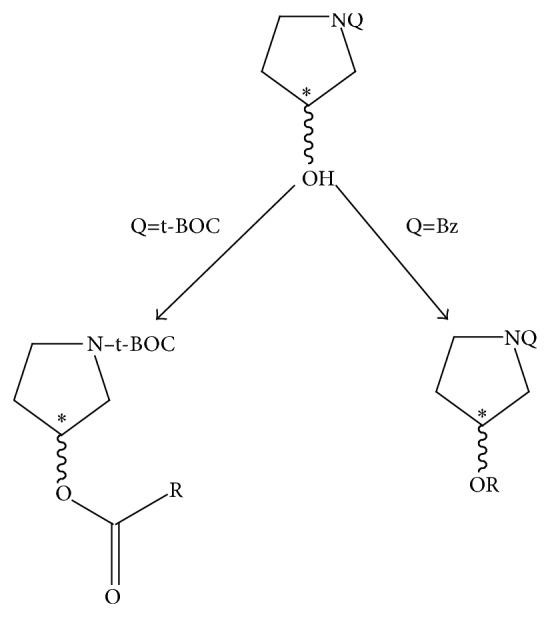


Armed with this information and using** 3** as a template, we sought to strip a great deal of the functional group and stereochemical complexity away from the molecule in order to see if significantly structurally simplified compounds prepared from readily commercially available starting materials could be engineered which would elicit a biological signal utilizing the novel proteasome assay developed in* C. elegans* described herein [28]. Based on this rationale, a N-substituted pyrrolidine framework was used which incorporated a stereogenic alcohol functional group handle at the same relative position of the sp^3^ ester oxygen in** 3** (Scheme 1). Mitsunobu-mediated acylation of the alcohol or S_N_2-mediated alkylation of the oxygen with ≥C-2 carbon chains provided simple derivatives with spatially comparable functional group density to** 3** without the four contiguous stereogenic centers.

Another primary design feature is the use of the 3-OH group to prepare electrophilic functional groups at C-3 relative to the ring nitrogen atom. The rationale behind this design element is the known mechanism of action of proteasome inhibitors containing electrophilic functionality. As previously indicated, the electrophilic nature of functional groups adjacent to the pyrrolidine ring is crucial to proteasome inhibition. Such well-known examples include vinyl sulfones [29], *α*′,*β*′-epoxyketones [30], and *β*-lactones [31]. These compounds act as irreversible inhibitors through the formation of a covalent bond with the active site Thr1O^*γ*^ of the *β*-subunits thereby leading to cell death [32–34]. For example, nucleophilic ring opening of the *β*-lactone moieties of** 3** and** 4** would generate an ester group at C-3. The analogs are summarized in Table 1.

### 2.2. Structure-Activity Relationships

The design set of compounds prepared possessed either ester functionality at C-3, ester functionality at C-3 with an electrophilic group (i.e., Michael acceptors), functional groups which might be converted to such groups through metabolic conversion, or electrophilic groups attached directly to C-3 of the pyrrolidine ring system. There are clear trends which were observed from the screening data. The most active compounds in the series proved to be those which could function as Michael acceptors or had the potential to be converted to electrophilic functional groups through oxidative metabolism (i.e., allyl ethers, epoxides). Compounds containing electrophilic groups attached directly to C-3 of the pyrrolidine ring were either inactive or weakly active at best. Dramatic differences between the* R-* and* S-*pyrrolidine core structures bearing the same functional groups were not observed for the limited number of compounds prepared for this study with the exception of** 4** compared to** 12**, both bulky t-BOC derivatives. The t-BOC group may serve a similar function to the hydroxyisobutyl side chain as previously described for** 3** to account for the observed activity of the* R-*isomer. N-Substitution appears to be critical for these compounds, with N-benzyl more active compared to the N-*t*-butyloxycarbonyl (**9** versus** 4**;** 12** versus** 16**). Future studies will address optimization of the pyrrolidine ring N-substituent and investigation of other C-3 electrophilic moieties.

### 2.3. Screen for Candidate Compounds

As our primary screen we assayed for compounds that were able to alter the expression of the GLD-1::GFP transgene in live animals similar to known proteasome inhibitors and RNAi inhibition of core proteasome subunits. In control animals GLD-1::GFP has a tightly restricted expression pattern that begins prior to the entry into meiosis, reaches maximal levels during the pachytene stage (meiotic prophase), and extends to the loop region (diplotene/diakinesis). Immediately after the loop region GLD-1 protein levels drop abruptly and are at very low levels during oogenesis (Figure 2(A)). In contrast, the* gld-1* mRNA is present throughout the germ line and is at high levels in oocytes even in the absence of detectable GLD-1 protein [19]. This dramatic downregulation of GLD-1 levels across a few cell diameters, even in the presence of high mRNA levels, suggests that the GLD-1 protein is actively removed prior to oocyte development. To identify control reagents we tested the known proteasome inhibitors epoxomicin, clasto-lactacystin *β*-lactone, lactacystin, MG115, and MG132 for the ability of phenocopy RNAi reduction-of-function in proteasome subunits in a* C. elegans*. Of the compounds tested, none were found to have any discernable effects that were similar to RNAi of proteasome subunits under standard culture conditions on NGM plates with OP50 bacteria. In liquid culture, however, we found epoxomicin and clasto-lactacystin *β*-lactone were able to alter the expression of a GLD-1::GFP transgene and phenocopy the effect of RNAi based reduction of proteasome function.

To identify compounds that could cause phenotypes indicative of loss of proteasome function, treated animals, and controls were scored at 2, 4, and 6 hours under the fluorescent dissecting scope for any changes in the GLD-1::GFP pattern relative to the control. All compounds screened were coded (single-blind) and scored by multiple individuals for activity. Compounds that resulted in any changes to GLD-1::GFP were fixed and stained to assess nuclear morphology and determine the extent of GLD-1::GFP misexpression. Phenotypes that scored as “positive” in this screen include increase in maximal levels of GLD-1::GFP levels, the ectopic expression of GLD-1::GFP in oocytes, and abnormal nuclear morphology (Figure 3, (B)–(D)). Compounds where toxicity and activity on GLD-1::GFP could not be separated were not pursued. Unlike control animals, GLD-1::GFP was detected beyond the loop region of treated animals with ectopic expression in oocytes. Of animals with increased GLD-1::GFP levels, the maximum fluorescent intensity in the pachytene region was found to be transiently higher in treated animals relative to controls (131.0 ± 8.9—versus—109.2 ± 6.4, *n* = 5) between 4 and 6 hours of treatment. As abnormal nuclear morphology and a general breakdown in germ line polarity were observed, the levels of GLD-1 were similar control animals (Figure 3, (D)). Treatment longer than 6 hours was attempted but abandoned due to abnormal morphology in the control after prolonged culture in liquid.

Taken together, the higher levels of GLD-1 and the misexpression of GLD-1 in oocytes suggest that normal turnover of the protein had been disrupted. Compounds 73T, 53P, 33K, and clasto-lactacystin *β*-lactone were similar in their ability to cause GLD-1::GFP misexpression; however, concentrations approximately 100-fold higher than epoxomicin were required in each case. Relative to tissue cultures based systems, the concentrations required to observe phenotypes in* C. elegans* in liquid were approximately 100–1000-fold higher depending on the assay [35, 36]. The higher concentrations are likely required due to the large number of transporters associated with multidrug resistance expressed in* C. elegans* [37]. We screened all compounds that resulted in the ectopic expression GLD-1::GFP transgene in the primary screen for the ability to increase the level of basal apoptosis in the* C. elegans* gem line.

### 2.4. Apoptosis

The primary mechanism by which proteasome inhibitors are able to restrict the cell division is the induction of apoptosis. The ectopic accumulation proteins caused by a block in proteasome function that are involved in the regulation of the cell cycle, such as cyclins and cyclin dependent kinase inhibitors, results in aberrant cell cycle progression and cell death [6]. In addition, as anticancer therapeutics, proteasome inhibitors such as Bortezomib have been demonstrated to improve the efficacy of standard cancer therapies to induce apoptosis in animal models [38]. To determine if the novel molecules synthesized for this work could induce apoptosis we used the* C. elegans* strain* bcIs39 *which expresses a functional CED-1::GFP fusion protein [39]. Unlike the deaths that occur during development of somatic lineages, programmed cell death in the germ line does not follow a set pattern determined by a fixed lineage. The vast majority of apoptosis in the germ line occurs at the loop region of the gonad where developing oocytes exit in the pachytene stage of meiotic prophase [40]. The CED-1::GFP fusion protein is functional and is expressed in the engulfing sheath cells that are required for the engulfment of neighboring cell corpses [41]. Essentially, the bcIs39 strains allows for the real-time visualization of cell corpses that are in the process of engulfment by scoring cells with “halos” (Figure 3).

**Figure 6:**
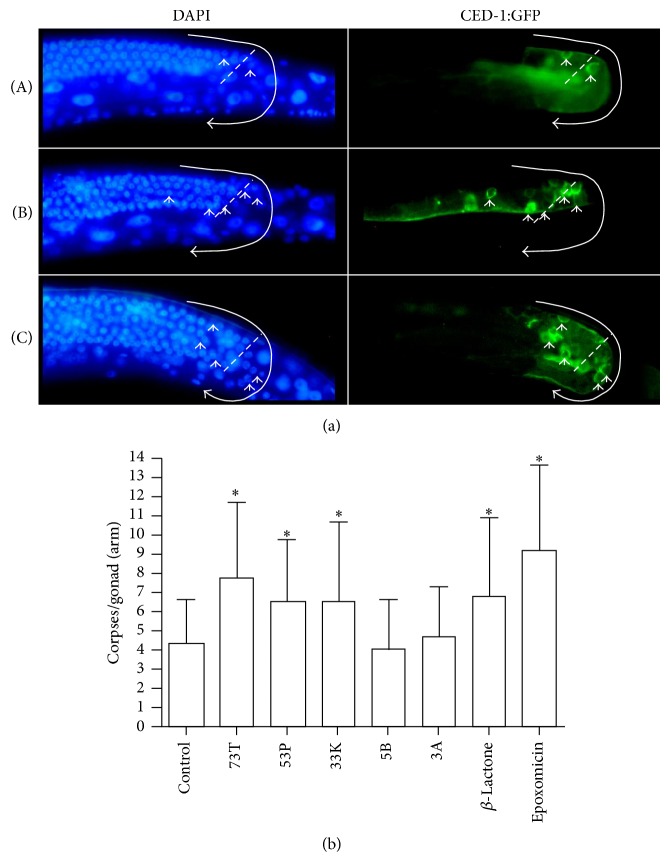
Inhibition of the proteasome increases germ line apoptosis in* C. elegans*. (a) Paired DAPI (nuclei, left) and CED-1::GFP (right) images from animals treated with carrier control (A), epoxomicin (B), or 73T (C). Representative examples of increased apoptosis (B and C) are shown where GFP halos are indicative of apoptosis, dashed line is at the loop region (pachytene-diplotene/diakinesis boundary), curved line ending in an arrow follows the germ line, and closed arrow heads are examples of apoptotic nuclei. Surface views of the germ line are shown; however, GFP halos in all focal planes were used in quantifying apoptosis (graph). All images are from 6 hours of treatment. (b) Compounds 73T, 53P, 33K, 5B, 3A, clasto-lactacystin *β*-lactone, and epoxomicin were tested for their ability to induce cell death in the* C. elegans* germ line over basal levels. The graph represents the average (one deviation indicated by the bar) of 50 gonad arms scored for each compound. Significance at *P* < 0.05 using the Mann-Whitney test indicated with “∗.”

To assess germ line apoptosis, treated and control animals were observed under the fluorescent dissecting for CED-1::GFP halos consistent with cells in the process of engulfment. Cell counts were performed on fixed animals on slides using a fluorescence compound scope. Similar to RNAi of core proteasome subunits, the known proteasome inhibitors clasto-lactacystin *β*-lactone and epoxomicin were able to induce apoptosis in the* C. elegans* germ line (Figure 6, graph). Longer incubations were attempted; however, control animals had widely varying numbers of germ line apoptotic cells after 10–12 hours; therefore, all treatments were limited to a maximum of six hours. Importantly, the numerical values obtained for apoptotic cells in control animals on OP50 seeded NGM plates and in liquid culture were similar to previously published results of approximately 2–6 apoptotic germ cells per gonad arm at six hours in culture (Figure 6, graph) [42–44].

## 3. Conclusion

In this work, we have described a screening protocol for compounds with characteristics common to proteasome inhibitors using a whole animal model system. The protocol offers good sensitivity yet allows for the rapid exclusion of compounds with low activity or nonspecific cytotoxicity. Importantly, this procedure was designed such that high throughput screening could be performed in 96-well format at very low reagent cost. While amenable to high throughput, one of the limits of this screening protocol is the inability to perform long-term treatments in liquid culture. A logical area for further development is to generate compounds that will also work on OP50 seeded agar plates such that longer treatments could be attempted.

A potential complication of our screening protocol is that the compounds assayed may be indirectly upregulating GLD-1::GFP. For example, disrupting the SCF complex or E3 responsible for polyubiquitination of GLD-1 may have similar phenotypes and result in a false positive. This caveat may also extend to negative regulators of other ubiquitin ligases and ubiquitin shuttling factors or cofactors, which could lead to an accumulation of GLD-1::GFP unrelated to 26S inhibition. In fact, it is formally possible that inhibition of a deubiquitylating enzyme could lead to the increase in GFP signal. We have used multiple phenotypes common to well-established 26S inhibitors as the primary diver for our screen; however, to formally address if any of the compounds are directly inhibiting the proteasome, a tertiary screening using cell-based or direct* in vitro* assays is advised. The next logical step will be to pursue these compounds (or their metabolites) as direct regulators of the proteasome. The further assessment of compounds identified using this screening procedure may prove useful in resolving the biological roles of the proteasome in a genetically tractable model system and feed into the development of therapeutic protocols in other systems.

## 4. Experimental Section

Reactions were monitored by TLC (thin layer chromatography) using precoated silica gel plates (glass or plastic) containing a fluorescent indicator. Detection was done by UV (254 nm), 1%, aqueous potassium permanganate solution, or I_2_ on silica gel. Anhydrous MgSO_4_ was used to dry organic solutions during workups, and the removal of solvents was carried out under vacuum with a rotary evaporator. Flash column chromatography was performed using silica gel 60 (230–400 mesh). Melting points were determined in a Mel-Temp heating block apparatus and are uncorrected. IR spectra were recorded on a Perkin-Elmer Model Spectrum 2000 FT-IR. GC/MS data were obtained from an Agilent Technologies 6850 GC/5973 MSD. ^1^H and ^13^C NMR spectra were recorded with a Varian Gemini 300 MHz nuclear magnetic resonance spectrometer referencing tetramethylsilane and utilizing CDCl_3_ lock. All the described compounds had ≥95% purity based on GC/MS analysis unless otherwise indicated.

### 4.1. General Procedure for the Acylation of Pyrrolidinols* via* the Mitsunobu Reaction: Method A [45]

A 250 mL three-necked round bottom flask equipped with a stir bar, N_2_ inlet, rubber septum, and thermometer was charged with anhydrous THF, the N-protected pyrrolidinol (1.0 equivalent), the acid (4.0 equivalents), and triphenylphosphine (4.0 equivalents). The resulting mixture was cooled in an ice bath under N_2_ and diethyl azodicarboxylate (4.0 equivalents; 40 wt% in toluene) was added dropwise at such a rate to maintain the temperature below 10°C. Upon completion of the addition, the reaction was allowed to warm to room temperature and stir overnight. The mixture was then heated at 40°C for* ca.* 4 h. The mixture was then poured into* t*-butyl methyl ether (50 mL) and the organics were washed with 5% NaHCO_3_ (3 × 30 mL). The combined aqueous washes were extracted with t-butyl methyl ether, the organics combined and dried (Na_2_SO_4_), filtered, and concentrated. The residue was diluted with t-butyl methyl ether (2 parts) followed by hexanes (1 part). The resulting suspension was filtered to remove solids and the filtrate was concentrated* in vacuo*. The residue was purified by flash chromatography eluting with 8% t-butyl methyl ether/hexanes to provide the ester.

### 4.2. General Procedure for the Acylation of the Pyrrolidinols with Anhydrides: Method B [46]

A mixture of the N-protected pyrrolidinol (5.0 equivalent), 4-(N,N-dimethylamino)pyridine, acetic anhydride (1.0 equivalent), and CH_2_Cl_2_ (10 mL) was stirred overnight at ambient temperature. The reaction mixture was diluted with 30 mL of CH_2_Cl_2_ and transferred to a separatory funnel. The mixture was washed with brine (2 × 30 mL) and the organics were dried (Na_2_SO_4_), filtered, and concentrated. The residue was purified by flash chromatography eluting with 8% t-butyl methyl ether/hexanes to provide the ester.

### 4.3. General Procedure for the Alkylation of the Pyrrolidinols: Method C [47]

A round bottom flask equipped with a stir bar and reflux condenser was charged with powdered NaOH (2.5 equivalents), H_2_O (5.6 equivalents), and the pyrrolidinol (1.0 equivalent). The mixture was warmed in a water bath to 60°C at which point tetrabutylammonium hydroxide (0.03 equivalents) and allyl bromide (1.75 equivalents) were added. The mixture was refluxed for 5 h and was allowed to stir at ambient temperature overnight. The biphasic mixture was separated and the aqueous phase was extracted with hexane (20 mL). The combined organics were washed with water (3 × 20 mL), dried over anhydrous MgSO_4_, and concentrated to afford the product which was used without further purification.

### 4.4. General Procedure for the Epoxidation of the Pyrrolidinol Vinyl Ethers: Method D [48]

A solution of the allyl ether (**6** or** 14)** in 5 mL CH_2_Cl_2_ (1.0 equivalents) was added dropwise to a solution of m-chloroperbenzoic acid (1.25 equivalents) in 10 mL CH_2_Cl_2_ at 0°C. The reaction mixture was stirred at 0°C for 1 h and then at room temperature for 16 h. Aliquots were drawn and analyzed by GC/MS. Additional m-chloroperbenzoic acid (1.25 equivalents) was added and the reaction stirred an additional 24 h. This was repeated until the reaction was determined to be complete by GC/MS. The reaction mixture was washed with NaHCO_3_ (2 × 30 mL), dried (MgSO_4_), filtered, and concentrated* in vacuo*.

### 4.5. General Procedure for the Sulfonylation of the Pyrrolidinols with Sulfonyl Chlorides: Method E [49]

To a solution of the N-protected pyrrolidinol (1.0 equivalent) in pyridine or triethylamine (20 mL) cooled to 0°C was added the sulfonyl chloride (1.2 equivalent) portionwise over 20 min. The mixture was allowed to warm to room temperature and was stirred overnight at ambient temperature. The reaction mixture was poured into a separatory funnel containing 100 mL 5% HCl. The mixture was extracted with ethyl acetate (3 × 30 mL). The combined organics were dried (MgSO_4_), filtered, and concentrated. The residue was purified by flash chromatography eluting with 25% EtOAc in hexanes.

(*R*)-*tert*-Butyl-3-(methylsulfonyl)pyrrolidine-1-carboxylate (**1**, Method E) was isolated as pale yellow oil using triethylamine as the base (>99%); ^1^H NMR *δ* 5.16 (m, 1H, diastereotopic ring 3-CH); 3.30–3.58 (m, 4H, ring 2 and 5-CH_2_); 2.92 (s, 3H, mesylate CH_3_); 1.96–2.15 (m, 2H, ring 4-CH_2_), 1.32 (s, 9H,* t*-BOC CH_3_); ^13^C NMR *δ* 28.4, 28.5, 32.0, 32.6, 38.6, 38.7, 43.7, 43.9, 46.2, 51.9, 52.2, 79.8, 80.4, and 154.2; IR (film) 3488, 2979, 1694, 1410, 1366, 1170, 1118, 957, 924, and 900 cm^−1^. EIMS (70 eV): 265 [M]^+^, 206 [M-*t*-Bu]^+^, and 192 [M-*t*-BuO]^+^.

(*S*)-*tert*-Butyl-3-(methylsulfonyl)pyrrolidine-1-carboxylate (**11**, Method E) was isolated as pale yellow oil using triethylamine as the base (>99%); ^1^H NMR *δ* 5.16 (m, 1H, diastereotopic ring 3-CH); 3.30–3.58 (m, 4H, ring 2 and 5-CH_2_); 2.32 (s, 3H, tosylate CH_3_); 1.82–2.10 (m, 2H, ring 4-CH_2_), 1.32 (s, 9H,* t*-BOC CH_3_); ^13^C NMR *δ* 28.4, 28.5, 32.0, 32.6, 38.6, 38.7, 43.7, 43.9, 46.2, 51.9, 52.2, 79.8, 80.4, and 154.2; IR (film) 3489, 2977, 1695, 1410, 1364, 1170, 1120, 953, 924, and 900 cm^−1^. EIMS (70 eV): 265 [M]^+^, 206 [M-*t*-Bu]^+^, and 192 [M-*t*-BuO]^+^.

(*R*)-*tert*-Butyl-3-tosylpyrrolidine-1-carboxylate(**2**, Method E) was isolated asa water-white oilusing pyridine as the base (16%); ^1^H NMR *δ* 7.67 (d, 2H, ArH); 7.38 (d, 2H, ArH); 4.92 (m, 1H, ring 3-CH); 3.32–3.42 (m, 4H, ring 2- and 5-CH_2_); 2.32 (s, 3H, ArCH_3_); 1.96–2.15 (m, 2H, ring 4-CH_2_), 1.32 (s, 9H,* t*-BOC CH_3_); EIMS (70 eV): 282 [M-*t*-Bu]^+^, 268 [M-*t*-BuO]^+^; 155 [C_7_H_7_SO_2_]^+^; 113 [C_5_H_7_NO_2_]^+^, 91 [C_7_H_7_]^+^; 57 [*t*-Bu]^+^.

(*R,  E*)-*tert*-Butyl-3-[(2-methylbut-2-enoyl)oxy]pyrrolidine-1-carboxylate (**3**, Method A) was isolated as a pale yellow oil (85.4%); ^1^H NMR *δ* 6.73 (q, 1H, vinylic CH); 4.11 (p, 1H, 3-O-CH); 3.43 (m, 2H, ring N-CH_2_); 3.31 (m, 2H, ring N-CH_2_); 1.93 (br m, 2H, diastereotopic ring 4-CH_2_); 1.78 (d, 3H, vinylic CH_3_); 1.68 (s, 3H, vinylic CH_3_); 1.33 (s, 9H;* t*-BOC CH_3_); ^13^C NMR *δ* 14.1, 14.3, 14.4, 14.6, 28.5, 30.9, 31.7, 43.6, 44.2, 72.7, 74.1, 79.6, 133.3, 133.5, 137.9, 138.1, 152.5, 154.6, 167.5, and 179.9; IR (film) 3155, 2984, 1692, 1469, 1382, 1261, 1165, and 1095 cm^−1^; EIMS (70 eV): 196 [M-*t*-BuO]^+^, 113 [C_5_H_7_NO_2_]^+^, and 83 [OCC_4_H_7_]^+^; 57 [*t*-Bu]^+^.

(*R*)-*tert*-Butyl-3-(acryloyloxy)pyrrolidine-1-carboxylate (**4**, Method A) was isolated as a pale yellow oil (54.4%); ^1^H NMR *δ* 6.30 (d, *J* = 5.8 Hz, 1H, vinylic CH); 5.99 (dd, *J* = 3.5, 5.8 Hz, 1H, vinylic CH); 5.73 (d, *J* = 3.5 Hz, 1H, vinylic CH); 5.23 (br m, 1H, ring diastereotopic 4-CH); 3.47 (m, 2H, N-CH_2_); 3.33 (m, 2H, N-CH_2_); 1.95 (m, 2H, ring 4-CH_2_); 1.34 (s, 9H,* t*-Bu CH_3_); ^13^C NMR *δ* 28.6, 30.9, 31.7, 43.7, 44.12, 51.5, 51.9, 73.3, 74.1, 79.7, 128.4, 131.4, 154.5, and 165.8; IR (film) 3061, 2977, 2885, 1727, 1620, 1479, 1412, 1366, 1297, 1270, 1192, 1117, and 1096 cm^−1^. EIMS (70 eV): 168 [M-*t*-BuO]^+^, 113 [C_5_H_7_NO_2_]^+^, and 57 [*t*-Bu]^+^.

(*S*)-*tert*-Butyl-3-(acryloyloxy)pyrrolidine-1-carboxylate (**12**, Method A) was isolated as a pale yellow oil (43.6%); ^1^H NMR *δ* 6.30 (d, *J* = 5.8 Hz, 1H, vinylic CH); 5.99 (dd, *J* = 3.5, 5.8 Hz, 1H, vinylic CH); 5.73 (d, *J* = 3.5 Hz, 1H, vinylic CH); 5.23 (br m, 1H, ring diastereotopic 4-CH); 3.47 (m, 2H, N-CH_2_); 3.33 (m, 2H, N-CH_2_); 1.95 (m, 2H, ring 4-CH_2_); 1.34 (s, 9H,* t*-BOC CH_3_); ^13^C NMR *δ* 28.6, 30.9, 31.7, 43.7, 44.12, 51.5, 51.9, 73.3, 74.1, 79.7, 128.4, 131.4, 154.5, and 165.8; IR (film) 3061, 2979, 2885, 1727, 1620, 1479, 1412, 1366, 1297, 1270, 1192, 1117, and 1096 cm^−1^; EIMS (70 eV): 168 [M-*t*-BuO]^+^, 113 [C_5_H_7_NO_2_]^+^, and 57 [*t*-Bu]^+^.

(*R*)-tert-Butyl-3-acetoxypyrrolidine-1-carboxylate (**5**, Method B) was isolated as a pale yellow oil (61.6%); ^1^H NMR *δ* 5.14 (br m; 1H, 1H, ring diastereotopic O-CH); 3.18–3.46 (br m, 4H, ring N-CH_2_); 1.92 (s, 3H, COCH_3_); 1.88 (partially obscured m, 2H, ring 4-CH_2_); 1.33 (s, 9H,* t*-BOC CH_3_); ^13^C NMR *δ* 21.2, 28.6, 30.8, 31.6, 43.7, 44.1, 51.5, 51.9, 73.1, 74.0, 79.6, 154.5, and 170.8; IR (film) 2978, 2885, 1741, 1698, 1406, 1366, 1245, 1169, 1116, and 1096 cm^−1^; EIMS (70 eV): 228 [M-H]^+^, 169 [M-CH_3_CO_2_H]^+^, 156 [M-*t*-BuO]^+^, 113 [C_5_H_7_NO_2_]^+^, and 57 [*t*-Bu]^+^.

(*S*)-*tert*-Butyl-3-acetoxypyrrolidine-1-carboxylate (**13**, Method B) was isolated as a pale yellow oil (94.2%); ^1^H NMR *δ* 5.14 (br m, 1H, ring diastereotopic O-CH); 3.18–3.46 (br m, 4H, ring N-CH_2_); 1.92 (s, 3H, COCH_3_); 1.88 (partially obscured m, 2H, ring 4-CH_2_); 1.33 (s, 9H,* t*-Bu CH_3_); ^13^C NMR *δ* 21.2, 28.6, 30.8, 31.6, 43.7, 44.1, 51.5, 51.9, 73.1, 74.0, 79.6, 154.5, and 170.8; IR (film) 2978, 2885, 1741, 1698, 1406, 1366, 1245, 1169, 1116, and 1096 cm^−1^; EIMS (70 eV): 228 [M-H]^+^, 169 [M-CH_3_CO_2_H]^+^, 156 [M-*t*-BuO]^+^, 113 [C_5_H_7_NO_2_]^+^, and 57 [*t*-Bu]^+^.

(*R*)-*tert*-Butyl-3-(allyloxy)pyrrolidine-1-carboxylate (**6**, Method C) was isolated as a clear oil (79.2%); ^1^H NMR *δ* 5.78 (m, 1H, vinylic CH); 5.10 (dd, *J* = 4.0 Hz, 9.8 Hz, 2H, vinylic CH_2_); 3.94 (m, 1H, ring diastereotopic O-CH); 3.84 (m, 2H, diastereotopic O-CH_2_); 3.30 (m, 4H, ring N-CH_2_); 1.84 (m, 2H, diastereotopic ring 4-CH_2_); 1.34 (s, 9H,* t*-BOC CH_3_); ^13^C NMR *δ* 28.7, 30.7, 31.7, 43.7, 44.2, 50.9, 51.6, 70.0, 79.3, 84.6, 117.2, 134.7, and 154.7; IR (film) 3081, 2977, 2933, 2884, 1698, 1478, 1455, 1365, 1254, 1169, 1118, and 1079 cm^−1^; EIMS (70 eV): 227 [M]^+^, 171 [M-C_4_H_8_]^+^, 156 [M-*t*-BuO]^+^, 113 [C_5_H_7_NO_2_]^+^, and 57 [*t*-Bu]^+^.

(*S*)-*tert*-Butyl-3-(allyloxy)pyrrolidine-1-carboxylate (**14**, Method C) was isolated as a clear oil (74.2%); ^1^H NMR *δ* 5.78 (m, 1H, vinylic CH); 5.10 (dd, *J* = 4.0 Hz, 9.8, 2H, vinylic CH_2_); 3.94 (m, 1H, ring diastereotopic O-CH); 3.84 (m, 2H, diastereotopic O-CH_2_); 3.30 (m, 4H, ring N-CH_2_); 1.84 (m, 2H, diastereotopic ring 4-CH_2_); 1.34 (s, 9H,* t*-BOC CH_3_); ^13^C NMR *δ* 28.7, 30.7, 31.7, 43.7, 44.2, 50.9, 51.6, 70.0, 79.3, 84.6, 117.2, 134.7, and 154.7; IR (film) 3081, 2977, 2933, 2884, 1698, 1478, 1455, 1365, 1254, 1169, 1118, and 1079 cm^−1^; EIMS (70 eV): 227 [M]^+^, 171 [M-C_4_H_8_]^+^, 156 [M-*t*-BuO]^+^, 113 [C_5_H_7_NO_2_]^+^, and 57 [*t*-Bu]^+^.

(*3R*)-*tert*-Butyl-3-(oxiran-2-ylmethoxy)pyrrolidine-1-carboxylate (**7**, Method D) was isolated as a clear oil (88.8%); ^1^H NMR *δ* 3.93 (m, 1H); 3.62 (m, 1H); 3.32 (m, 5H); 3.02 (m, 1H); 2.69 (m, 1H); 2.50 (m, 1H); 1.86 (m, 2H, diastereotopic ring 4-CH_2_); 1.48 (s, 9H,* t*-BOC CH_3_); ^13^C NMR *δ* 28.6, 30.4, 31.7, 43.7, 44.1, 44.4, 51.0, 69.7, and 79.4; 154.7; IR (film) 2977, 2933, 2886, 1696, 1478, 1408, 1367, 1252, 1220, 1168, and 1119 cm^−1^; EIMS (70 eV): 243 [M]^+^, 186 [M-C_4_H_7_]^+^, and 170 [M-*t*-BuO]^+^; 142 [M-*t*-BuOCO]^+^; 113 [C_5_H_7_NO_2_]^+^, 57 [*t*-Bu]^+^; purity = 93%.

(*3S*)-*tert*-Butyl-3-(oxiran-2-ylmethoxy)pyrrolidine-1-carboxylate (**15**, Method D) was isolated as a clear oil (>99%); ^1^H NMR *δ* 3.93 (m, 1H); 3.62 (m, 1H); 3.32 (m, 5H); 3.02 (m, 1H); 2.69 (m, 1H); 2.50 (m, 1H); 1.86 (m, 2H, diastereotopic ring 4-CH_2_); 1.48 (s, 9H,* t*-BOC CH_3_); ^13^C NMR *δ* 28.6, 30.4, 31.7, 43.7, 44.1, 44.4, 51.0, 69.7, and 79.4; 154.7; IR (film) 2977, 2933, 2886, 1696, 1478, 1408, 1367, 1252, 1220, 1168, and 1119 cm^−1^; EIMS (70 eV): 243 [M]^+^, 186 [M-C_4_H_7_]^+^, and 170 [M-*t*-BuO]^+^; 142 [M-*t*-BuOCO]^+^; 113 [C_5_H_7_NO_2_]^+^, 57 [*t*-Bu]^+^; purity = 92%.

(*R*)-1-Benzylpyrrolidin-3-yl 3-methylbut-2-enoate (**8**, Method A) was isolated as a pale yellow oil (48.3%); ^1^H NMR *δ* 7.06–7.25 (m, 5H, ArH); 5.60 (s, 1H, vinylic CH); 5.12 (m, 1H, diastereotopic ring 3-CH); 3.60 (d, 1H, diastereotopic benzylic CH_2_); 3.52 (d, 1H, diastereotopic benzylic CH_2_); 2.70 (m, 2H, ring N-CH_2_); 2.52 (m, 3H, ring N-CH_2_); 2.09 (m, 1H, ring diastereotopic CH); ^13^C NMR *δ* 20.8, 27.5, 32.0, 52.7, 60.3, 61.8, 73.5, 116.2, 127.2, 128.8, 138.5, 157.0, and 166.6; IR (film) 3085, 3062, 3028, 2915, 1714, 1651, 1494, 1444, 1378, 1350, 1271, and 1147 cm^−1^. EIMS (70 eV): 259 [M]^+^, 159 [M-C_3_H_4_O_2_]^+^, and 91 [C_7_H_7_]^+^.

(*R*)-1-Benzylpyrrolidin-3-yl acrylate (**9**, Method A) was isolated as pale yellow oil (71.9%); ^1^H NMR *δ* 7.06–7.20 (m, 5H, ArH); 6.24 (d, *J* = 5.9 Hz, 1H, vinylic CH); 6.05 (dd, *J* = 3.5, 5.9 Hz, 1H, vinylic CH); 5.70 (d, *J* = 3.5 Hz, 1H, vinylic CH); 5.20 (m, 1H, diastereotopic ring 3-CH); 3.60 (d, 1H, diastereotopic benzylic CH_2_); 3.52 (d, 1H, diastereotopic benzylic CH_2_); 2.70 (m, 2H, ring N-CH_2_); 2.50 (m, 2H, ring N-CH_2_); 1.96 (m, 2H, ring CH_2_); ^13^C NMR *δ* 32.0, 52.8, 60.0, 60.3, 74.4, 127.2, 128.4, 128.7, 129.0, 130.8, 138.6, and 166.2; IR (film) 3063, 3029, 2966, 1721, 1636, 1619, 1495, 1477, 1455, 1442, 1407, and 1201 cm^−1^; EIMS (70 eV): 230 [M-H]^+^, 159 [M-C_3_H_4_O_2_]^+^, and 91 [C_7_H_7_]^+^.

(*S*)-1-Benzylpyrrolidin-3-yl acrylate (**16**, Method A) was isolated as pale yellow oil (60.2%); ^1^H NMR *δ* 7.06–7.20 (m, 5H, ArH); 6.24 (d, *J* = 5.9 Hz, 1H, vinylic CH); 6.05 (dd, *J* = 3.5, 5.9 Hz, 1H, vinylic CH); 5.70 (d, *J* = 3.5 Hz, 1H, vinylic CH); 5.20 (m, 1H, diastereotopic ring 3-CH); 3.60 (d, 1H, diastereotopic benzylic CH_2_); 3.52 (d, 1H, diastereotopic benzylic CH_2_); 2.70 (m, 2H, ring N-CH_2_); 2.50 (m, 2H, ring N-CH_2_); 1.96 (m, 2H, ring CH_2_); ^13^C NMR *δ* 32.0, 52.8, 60.0, 60.3, 74.4, 127.2, 128.4, 128.7, 129.0, 130.8, 138.6, and 166.2; IR (film) 3063, 3029, 2966, 1721, 1636, 1619, 1495, 1477, 1455, 1442, 1407, and 1201 cm^−1^; EIMS (70 eV): 230 [M-H]^+^, 159 [M-C_3_H_4_O_2_]^+^, and 91 [C_7_H_7_]^+^.

(*R, E*)-1-Benzylpyrrolidin-3-yl 2-methylbut-2-enoate (**10**, Method A) was isolated as pale yellow oil (95.4%); ^1^H NMR *δ* 7.08–7.22 (m, 5H, ArH); 6.72 (q, 1H, vinylic CH); 5.09 (m, 1H, diastereotopic ring 3-CH); 3.58 (d, 1H, diastereotopic benzylic CH_2_); 3.50 (d, 1H, diastereotopic benzylic CH_2_); 2.79 (dd, 1H, ring N-CH); 2.64 (dd, 1H, ring N-CH); 2.52 (dd, 1H, ring N-CH); 2.41 (distorted dd, 1H, N-CH); 2.14 (m, 1H, diastereotopic ring 4-CH); 1.75 (m, 1H, diastereotopic ring 4-CH); 1.68 (s, 3H, vinylic CH_3_); 1.63 (d, 3H, vinylic CH_3_); ^13^C NMR *δ* 12.9, 14.5, 32.0, 52.7, 60.5, 63.8, 74.2, 127.2, 128.4, 128.7, 136.8, and 168.0; IR (film) 3086, 3062, 3027, 2826, 1757, 1706, 1650, 1605, 1494, 1453, 1443, 1371, 1347, and 1255 cm^−1^; EIMS (70 eV): 230 [M-H]^+^, 159 [M-C_3_H_4_O_2_]^+^, and 91 [C_7_H_7_]^+^.

### 4.6. Worm Strains

The strain* rrf-1(pk1417)(I);ozIs2(II)* was generated by using ballistic transformation of* unc-119(ed3)* with the MM016 and genomic a* gld-1::GFP* fusion [50]. The integrated sequence was mapped to chromosome II, outcrossed four times, and crossed to* rrf-1(pk1417) *to generate the* rrf-1(pk1417)(I);ozIs2(II)*. The* rrf-1(pk1417) *mutation confers somatic RNAi resistance and is not required for the chemical screen but is required to screen for germ line specific screens with RNAi to avoid lethality associated with depletion of proteasome components in somatic tissues. This integrated transgene has the same expression pattern and endogenous GLD-1 (Figure 1, top), has similar levels of expression to endogenous GLD-1 (Figure 3(b)), and rescues the* gld-1(null)* tumorous phenotype indicating that it is fully functional. The ability of the transgene to rescue the* gld-1(q485)* (null) phenotype was assessing by crossing* ozIs2(II)* males were crossed to the* gld-1(q485)/dpy-5(e61)unc-13(e51)* balanced strain and non-*dpy* non-*unc* with GFP expression were selected. The presence of the* gld-1(q485)* allele was confirmed by sequencing and outcrossing to N2 to recover the* q485* germ line tumor phenotype in non-GFP animals. The* ozIs2(II)* was able to rescue the* gld-1(q485) *germ line tumor phenotype in 100% of animals. Strain MD701* bcIs39[P(lim-7)ced-1::GFP + lin-15(+)](V)* which expresses a functional CED-1::GFP fusion protein in the sheath cells for analysis of apoptosis was obtained from* Caenorhabditis* Genetics Center (CGC, University of Minnesota, Minneapolis, MN 55455, USA) [39].

### 4.7. Western Blot

Either 25 or 50 worms were picked directly into 1.5 mL centrifuge tubes in 1 mL of PBS and allowed to sit for 5 min to clear bacteria from the gut. Worms were then washed 3 times with 1 mL of PBS using a microcentrifuge (1000 ×g, 3 min). The final wash aspirated with a drawn Pasteur pipet and 20 *μ*L of 2X a modified SDS sample buffer (20% glycerol, 100 mM Tris-HCL pH = 6.8, 4% SDS, 0.01 mg/mL bromophenol blue, 1% *β*-mercaptoethanol) added directly to the worm “pellet” and boiled for 5 min. The insoluble debris was removed by centrifugation at 14,000 ×g for 10 min at 4°C and the entire supernatant for each sample was loaded on a discontinuous SDS-PAGE gel (3.5% stacking, 10% resolving). Proteins were transferred to Hybond PVDF (polyvinylidene fluoride, RPN303F, GE Healthcare, USA) membrane using semidry transfer and blocked in blocking buffer (25 mM Tris pH = 8, 125 mM NaCl, 0.1% Tween 20, 5% nonfat dry milk) overnight at 4°C with gentile agitation. Membranes were probed with affinity purified anti-GLD-1 antisera [19] diluted 1 : 50 in blocking buffer with overnight incubation with gentile agitation. After extensive washing in PBS, membranes were incubated for 4 hours in anti-rabbit peroxidase-conjugated secondary antibody (711-035-152, Jackson ImmunoResearch, USA) in blocking buffer. GLD-1 protein was detected using Amersham ECL Plus (RPN2209, GE Healthcare, USA).

### 4.8. Worm Culture and Assay

All stock cultures were maintained on* Escherichia coli* OP50 on NGM plates at 20°C as previously described [51]. Both the commercially available proteasome inhibitors tested and potential inhibitors reported in this work were not effective on NGM plates (seeded or unseeded). There are several potential problems with drug delivery using standard culture techniques with live bacteria (OP50) as a food source. First, it is possible that the live bacteria may be processing the compounds and reducing activity. Second,* C. elegans* has the potential to code for 60 ABC transporters associated with multidrug resistance relative to 57 in Drosophila, 49 in humans, and 30 in yeast, a feature which is likely to make treatment problematic due to high rates of excretion [37]. To circumvent these issues we have used short-term liquid culture of* C. elegans* to greatly reduce the influence of bacteria and allow for the delivery of compounds at extremely high concentrations. Worms used for screening were collected at the L4 stage, matured to gravid adults (24 hours), and washed three times prior to liquid culture in PBS (137 mM NaCl, 12 mM Phosphate, 2.7 mM KCl, pH 7.4) using 96-well plates (Corning, Corning #9018, flat bottom polystyrene). To avoid any problems with abnormal morphology from long-term liquid culture all assays were for a maximum of 6 hours in duration where untreated and carrier controls had germ line nuclear morphology that were indistinguishable from worms cultured on OP50 seeded NGM plates. For first-pass analysis of novel compounds 10-fold serial dilutions of 100 mg/mL stocks in DMSO were used to roughly determine effective concentrations and eliminate compounds with high levels of toxicity. Throughout the duration of the assay worms were assayed for GFP and normal morphology at 2, 4, and 6 hours using a Leica MZ 16 FA Fluo Combi III. LC_50_ and 95% confidence intervals were determined by using the nonparametric Spearman-Karber method. For LC_50_ calculations mortality was defined as worms that were nonresponsive to head stimulation after tow hour recovery on OP50 seeded NGM plates. All chemicals, including previously defined proteasome inhibitors, were purchased from Sigma Aldrich (St. Louis, MO) or Cayman Chemical Company (Ann Arbor, MI) and used without further purification.

### 4.9. DAPI Staining and Apoptosis Assay

DAPI staining and apoptotic nuclei were scored as previously described [13, 41, 52]. Briefly, for whole* C. elegans* DAPI staining to visualize nuclei, animals were fixed and permeabilized with 100% methanol at −20°C for 5 min, washed in PBT (PBS + 0.1% Tween 20) three times, and stained using 100 ng/mL DAPI in PBT prior to mounting on 2% agar pads on microscope slides. Worms were either processed in directly in 96-well plates or 1.6 mL microcentrifuge tubes. Apoptotic cells were scored by counting GFP “halos” of CED-1::GFP (see strains) on the engulfing sheath cell in all focal planes after 6 hours of treatment. Examples are indicated on Figure 3. Longer term cultures, such as 12 hours and overnight, were attempted; however, an overall increase in apoptosis was observed starting with 12 hours in liquid compromising signal to noise in the assay. Significance at 0.05 for the apoptosis assay was determined by comparison of treated and untreated samples using the Mann-Whitney test. All epifluorescent images were captured with a Nikon Eclipse E800 with ACT-1 (v2.62) software and processed with Pixelmator 1.4.1 (Pixelmator Team Ltd., London, UK). All image postprocessing functions (brightness, contrast, pseudocolor, unsharp mask) were performed identically for GFP images. MFI and maxima measurements were calculated suing Image J and represent the average of 5 arms [53]. DAPI images were processed to reveal the maximum number of nuclei and processed separately.

## Figures and Tables

**Table 1:** N-t-BOC and N-benzyl 3-hydroxypyrrolidine analogs prepared.

Compound	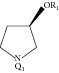		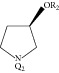		EC_50 _(mM)
	R_1_	Q_1_	R_2_	Q_2_	
**1**	SO_2_CH_3_	t-BOC			N/A
**2**	SO_2_(4-CH_3_C_6_H_4_)	t-BOC			141.7
**3**	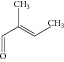	t-BOC			13.7
**4**	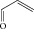	t-BOC			15.3
**5**	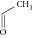	t-BOC			N/A
**6**	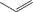	t-BOC			73.0
**7**	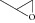	t-BOC			114.6
**8**	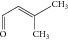	Bz			9.0
**9**	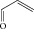	Bz			5.1
**10**	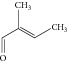	Bz			N/A
**11**			SO_2_CH_3_	t-BOC	N/A
**12**			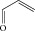	t-BOC	N/A
**13**			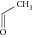	t-BOC	N/A
**14**			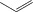	t-BOC	87.1
**15**			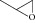	t-BOC	52.4
**16**			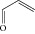	Bz	7.3
Epoxomicin					0.6
Omuralide					47
